# Metabolism of oxyfluorfen by actinobacteria *Micrococcus* sp. F3Y

**DOI:** 10.3389/fmicb.2025.1599015

**Published:** 2025-05-09

**Authors:** Li Yao, Yue Wen, Yuting Sha, Leqin Wang, Xianrui Bi, Shuhan Si, Min Shen, Shusong Zhang, Haiyan Ni

**Affiliations:** ^1^College of Marine and Bio–Engineering, Yancheng Teachers University, Yancheng, China; ^2^College of Biotechnology and Pharmaceutical Engineering, Nanjing Tech University, Nanjing, China; ^3^Nanchang Key Laboratory of Microbial Resources Exploitation & Utilization from Poyang Lake Wetland, College of Life Sciences, Jiangxi Normal University, Nanchang, China

**Keywords:** oxyfluorfen biodegradation, *Micrococcus* sp. F3Y, metabolic pathway, *pao* gene cluster, bioremediation

## Abstract

Oxyfluorfen, a potent diphenyl ether herbicide, has raised significant environmental concerns due to its persistence, toxicity to non‒target organisms, and potential carcinogenicity. Microbial degradation plays a crucial role in mitigating its impact, yet complete mineralization pathways remain poorly understood. In this study, we isolated *Micrococcus* sp. F3Y, an oxyfluorfen‒degrading actinobacterium, and evaluated its degradation efficiency in yeast powder‒supplemented mineral medium (YPM) medium and oxyfluorfen‒contaminated soil. Optimal conditions, pH, temperature, initial optical density (OD_600nm_) were determined. Metabolites were analyzed via UPLC/Q‒TOF MS, and a putative gene cluster was identified through draft genome sequencing. Strain F3Y completely degraded 100 mg/L oxyfluorfen within 12 h under optimal conditions (pH 7.0, 30°C, OD600=2.0), maintaining over 55% efficiency at 25‒42°C and above 62% across a pH range of 6.5‒8.0. When the initial oxyfluorfen concentration was ≤150 mg/L, the degradation rate exceeded 74%. Moreover, in oxyfluorfen‒contaminated soil (0.06 mg/kg), inoculation with strain F3Y restored soybean (*Glycine max*) growth, increasing shoot length from 4.22 cm (severely inhibited) to 28.8 cm, a nearly 7‒fold improvement. Additionally, F3Y achieved 98.2% degradation of oxyfluorfen (50 mg/kg) within 25 d in unsterilized soil. Eleven metabolites, including six new intermediates, were identified. Based on these, two novel degradation pathways were proposed: one initiated by nitro reduction and the other by diaryl ether cleavage. Both pathways culminated in aromatic ring opening and complete mineralization. In addition, a potential 24.3 kb gene cluster, *pao*, was suggested. Comprising thirteen genes, it was hypothesized to participate in the ring cleavage of intermediate products during oxyfluorfen degradation. This study provided the first comprehensive evidence of *Micrococcus* mediated oxyfluorfen mineralization, offering new insights into actinobacterial metabolic versatility. With its high degradation efficiency, environmental resilience, and detoxification ability, F3Y was an ideal candidate for bioremediation. These finding not only enhanced the understanding of herbicide degradation but also provided a sustainable solution to address oxyfluorfen contamination in agricultural and natural ecosystems.

## 1 Introduction

Oxyfluorfen is a selective pre– and postemergent diphenyl ether herbicide, commonly utilized for controlling annual broad–leaved weeds and grasses in crops such as rice, corn, cotton, soybean, peanuts, vegetable gardens, and orchards (Li et al., 2022; Galvin et al., 2022; Huang et al., 2016). The herbicidal activity of oxyfluorfen is attributed to its inhibition of protoporphyrinogen oxidase (PPO), which disrupts chlorophyll synthesis and hampers photosynthesis in plants (Nandihalli et al., 1992; Phung and Jung, 2015). Its ultralow dosage requirement, high efficacy, long shelf life, and compatibility with various other herbicides make oxyfluorfen a cost–effective solution (Li et al., 2022; Zhao et al., 2016). However, the environmental persistence of oxyfluorfen presents significant threats to both ecosystems and human health (Majid et al., 2024; Chen et al., 2023; Mantzos et al., 2013; Mohamed et al., 2011). For instance, Hussein et al. (2012) indicated that oxyfluorfen may lead to a decrease or increase in the number of certain microorganisms, alter the diversity of microbial communities, and affect the metabolic activity of microorganisms in loam and sandy loam soils; Tejada et al. (2022) found that oxyfluorfen could reduce the relative abundance of Firmicutes and Acidobacteria in soil; Huang et al. (2022) reported that oxyfluorfen disrupts zebrafish cellular migration and induces oxidative stress and apoptosis; Bonnar and Tjeerdema (2025) showed that oxyfluorfen accumulates significantly in Californian rice paddy sediments, degrading slowly under anaerobic conditions (half–life >600 days), thereby elevating risks to benthic invertebrates and algal communities. Furthermore, emerging toxicological evidence has identified oxyfluorfen as an endocrine-disrupting compound. Murr et al. (2025) revealed that prepubescent male rats exposed to oxyfluorfen exhibited marked suppression of thyroid hormone synthesis, accompanied by delayed puberty onset, specifically, a 1.3–3.5–day deferral in preputial separation (a validated marker of rodent puberty), and reduced growth of androgen-sensitive tissues, including the prostate and seminal vesicles. These findings align with the U.S. EPA’s classification of oxyfluorfen as a probable human carcinogen due to its documented carcinogenic potential (Carmichael et al., 2016). Collectively, these findings highlight the critical need for developing efficacious remediation strategies to attenuate oxyfluorfen residues in ecosystems and mitigate their adverse environmental and health impacts.

Microbial degradation has gained significant recognition as an effective approach for oxyfluorfen remediation, owing to its superior efficiency, environmental compatibility, and economic viability (Gao et al., 2024; Zhao et al., 2024). To date, several microbial strains with oxyfluorfen–degrading capabilities have been identified, such as *Bacillus licheniformis* ATCC 21415 (Tejada et al., 2022), *Bacillus methylotrophicus* A1 and *Streptomyces* sp. B1 (Bhatt, 2017), *Chryseobacterium aquifrigidense* R21 (Zhao et al., 2016), *Sphingomonas wittichii* RW1 (Keum et al., 2008), and *Azotobacter* sp. (Chakraborty et al., 2002). These comprehensive studies have elucidated specific metabolites and proposed corresponding metabolic pathways. However, although these strains demonstrated distinct degradation routes, they all shared a common shortcoming in attaining complete mineralization. That is, none of them could fully transform oxyfluorfen into inorganic end products. While Chen et al. (2025) have identified cytochrome P450, methyltransferases, glycosyltransferases, and acetyltransferases as key enzymes participants in oxyfluorfen degradation in rice (*Oryza sativa*), and Bhatt (2017) revealed *B methylotrophicus* A1 and *Streptomyces* sp. B1 degrade oxyfluorfen via laccase and esterase, respectively, the molecular mechanisms governing microbial degradation of this herbicide remained largely elusive. Significant gaps persist in our understanding of both the enzymatic pathways involved and their associated genetic regulation mechanisms.

*Micrococcus*, a genus of gram–positive, non–spore–forming actinomycetes within the family *Micrococcaceae*, demonstrates remarkable environmental ubiquity, colonizing diverse habitats ranging from activated sludge and soil ecosystems to human skin microbiota, cryospheric environments, dairy waste systems, and atmospheric samples. Although nine distinct species have been taxonomically classified within this genus, few of them have demonstrated significant catabolic potential towards both natural and xenobiotic compounds (Lee et al., 2022). In this study, the strain *Micrococcus* sp. F3Y, capable of utilizing oxyfluorfen as its sole energy source for growth and completely degraded it, was isolated from activated sludge. The underlying degradation mechanisms were elucidated based on the comprehensive analysis of intermediate metabolites and genomic prediction of potential catabolic genes. Furthermore, the bioremediation potential of strain F3Y in oxyfluorfen–contaminated soil systems was evaluated by employing soybean plants as biological indicators to assess detoxification efficiency. This investigation not only expanded the known microbial resources for oxyfluorfen degradation but also provided novel insights into the metabolic versatility of the *Micrococcus* genus in xenobiotic compound transformation.

## 2 Materials and methods

### 2.1 Chemicals and media

Oxyfluorfen (>98% purity) was purchased from Sigma–Aldrich Chemical Co., Ltd. (Shanghai, China). Methanol and acetonitrile (HPLC grade) were purchased from Tedia Co., Ltd. (United States). Formic acid (HPLC grade) was purchased from Feipu Chemical Reagent Factory (Yancheng, China). Molecular biological reagents were purchased from TaKara Biotechnology Co., Ltd. (Dalian, China) and Kingsley Biotechnology Co., Ltd. (Nanjing, China) respectively. All other reagents used in this study were of analytical reagent grade. The Luria–Bertani medium (LB) contained 5.0 g/L NaCl, 10.0 g/L peptone, and 5.0 g/L yeast extract, pH 7.0. The mineral salt media (MSM) contained 1.0 g/L NH_4_NO_3_, 1.5 g/L K_2_HPO_4_, 0.5 g/L KH_2_PO_4_, 1.0 g/L NaCl, and 0.2 g/L MgSO_4_, pH 7.0. The solid media plates were prepared by adding 1.5–2% wt vol^−1^ agar to the above liquid media.

### 2.2 Isolation and identification of oxyfluorfen–degrading bacteria

The oxyfluorfen–degrading bacteria were screened by an enrichment method. An activated sludge sample was collected from the aerobic tank of a wastewater treatment system at an oxyfluorfen production facility (32.54°N, 121.07°E) located in Nantong City, Jiangsu Province, China. 10 g of the sample was added to 100 mL of MSM medium with the addition of 50 mg/L oxyfluorfen. The mixture was incubated at 30°C on a rotary shaker at 160 rpm for 7 d. The enrichments were performed over five rounds, and the concentration of oxyfluorfen increased by 10 mg/L each round. The enrichment solution with degradation effects was subsequently serially diluted, spread onto LB agar plates, and cultured at 30°C for 3 d. The different single colonies were picked, purified by the spread plate method. The oxyfluorfen–degrading abilities of the strains were detected using a UV–1800 spectrophotometer (Shimadzu, Japan). Initially, the strains were cultured in LB liquid medium at 30°C until they reached the logarithmic growth phase. Subsequently, the cells were harvested via low–speed centrifugation at 5000 × *g*. The harvested strains were washed twice and resuspended in normal saline to serve as inoculum. Equal volumes of the inoculum, all with the same initial OD_600nm_ value, were inoculated into MSM medium containing 100 mg/L oxyfluorfen. Triplicate cultures were incubated at 30°C with constant agitation at 150 rpm. A control group without bacterial inoculation was included to account for abiotic degradation processes. After 24 h of incubation, the culture filtrates were collected and extracted with an equal volume of dichloromethane. The mixture was shaken for 20 min, and then the lower organic phase was separated, dried over anhydrous Na_2_SO_4_, and analyzed using the UV spectrophotometer within a scan range of 180–400 nm. The oxyfluorfen–degrading ability of each strain was evaluated by comparing the absorbance changes of the samples from different strains with that of the control sample. Based on these results, a pure strain named F3Y, which exhibited the highest degradation efficiency, was selected for further investigation.

The morphology of F3Y cells was observed via transmission electron microscopy (H–7650; Hitachi). 16S rRNA gene sequence analysis and identification of the bacterium were performed as described by Lee et al. (2023). The genomic DNA was extracted from strain F3Y via a bacterial genomic DNA fast extraction kit (Takara) following the manufacturer’s instructions. The universal bacterial primer pair 27F and 1492R was used to amplify the 16S rRNA gene (Lane, 1991). The obtained 16S rRNA gene sequence (GenBank accession no. OQ933129) was compared with closely related sequences of reference organisms via the EzBioCloud identification service^1^ (Yoon et al., 2017). A phylogenetic tree was constructed with MEGA 7.0 via the neighbor–joining (NJ) method (Kumar et al., 2016).

### 2.3 Biodegradation characteristics of oxyfluorfen by strain F3Y

Strain F3Y was prepared into seed liquid using the same method detailed in the previous section. For the kinetic experiment, the seed culture was inoculated to MSM medium (100 mL) or YPM (MSM medium supplemented with yeast powder, 5 g yeast powder/1000 mL of MSM medium, 100 mL). The two media were both contained 100 mg/L oxyfluorfen. The initial OD_600nm_ of strain F3Y was 2.0, while inactivated strain, prepared via autoclaving (121°C, 20 min) were served as the control. All three replicate treatments were cultured at 150 rpm and 30°C. The growth of strain and the concentration of residual oxyfluorfen were detected every 2 h. The degradation and growth curves were fitted to a modified Gompertz model (Zwietering et al., 1990) using GraphPad Prism 8.0. The model equation of degradation was: Y = A. exp.{−exp [1 + μm.exp. (1). (*λ*–t)/A]}. Y was the percentage of degradation (%), t was the time (h), μm was the maximum degradation rate (% h-1), A was the maximum percentage of degradation (%), and λ was the lag time (h). The parameters were validated using a Student’s t test (*p* < 0.005).

The effects of environmental factors, including pH (5.0–9.0), temperature (25–50°C), initial oxyfluorfen concentration (50–300 mg/L), and initial cell density (OD_600nm_ = 0.5–3.0) on the oxyfluorfen–degrading ability of strain F3Y were determined according to the method described by Yao et al. (2015). The experimental pH and temperature ranges were selected to reflect typical agricultural soil profiles while encompassing the operational thresholds of strain F3Y observed during preliminary characterization. The kinetics parameters were subjected to a single–factor analysis of variance (one–way ANOVA) followed by a Fisher procedure (n = 3, *p* < 0.001) (GraphPad Prism 8.0). The influences of each environmental condition on oxyfluorfen degradation was evaluated under optimal degradation conditions with respect to the other environmental variables. The remaining concentration of oxyfluorfen was estimated via HPLC with a hypersil C–18 column (4.6 × 250 mm, 5 μm particle size) at 45°C. The mobile phase of organic phase sample was acetonitrile: distilled water with 5% acetic acid (90:10, v/v), and the mobile phase of the water phase solution was 0.01% formic acid: 0.01% acetonitrile (5:95, v/v). The detection wavelength was 254 nm. The flow rate was 1.0 mL/min, and the injection volume was 20 μL. The run time was 15 min.

### 2.4 Bioremediation potential of strain F3Y in oxyfluorfen–contaminated soil

The bioremediation efficacy of strain F3Y in oxyfluorfen–contaminated soil systems was evaluated through comprehensive analysis of morphological and physiological parameters of soybean (*Glycine max*) under controlled greenhouse conditions. The experimental soil was collected from the top 0–20 cm of the experimental field at Yancheng Teachers University (Yancheng, China), which had not been exposed to oxyfluorfen or other diphenyl ether herbicides in the past 5 years. The soil samples were sieved through a 2–mm mesh, air–dried, and then assigned to six distinct experimental groups: (1) Negative control (−oxyfluorfen, –F3Y), soil without oxyfluorfen and F3Y inoculation, used to assess the impact of basal growth conditions on soybean growth; (2) Oxyfluorfen toxicity assessment (+oxyfluorfen, –F3Y), soil amended with 0.06 mg/kg oxyfluorfen, used to evaluate the toxic effects of oxyfluorfen on soybean growth; (3) Inactivated F3Y impact (−oxyfluorfen, +inactivated F3Y), soil inoculated with inactivated F3Y strain, employed to determine the impact of the inactivated strain itself on soybean growth; (4) Inactivated F3Y in oxyfluorfen–contaminated soil (+oxyfluorfen, +inactivated F3Y), soil containing both 0.06 mg/kg oxyfluorfen and inactivated F3Y strain, utilized to evaluate the impact of the inactivated F3Y strain on soybean growth in the presence of oxyfluorfen; (5) Viable F3Y impact (−oxyfluorfen, +F3Y), oxyfluorfen–free soil inoculated with viable F3Y strain, served to assess the influence of the viable F3Y strain itself on soybean growth; and (6) Bioremediation potential assessment (+oxyfluorfen, +F3Y), soil treated with both 0.06 mg/kg oxyfluorfen and viable F3Y strain, functioned as the experimental group to evaluate the bioremediation potential of the viable F3Y strain in oxyfluorfen–contaminated soil. The prepared strain F3Y suspension, as described in the preceding section, was inoculated into the treated soil at a concentration of 5% (v/w) (Zhao et al., 2022). 1 kg aliquots of the treated soil were transferred to standardized pots (13 cm upper diameter × 10 cm lower diameter × 12 cm height). Soybean seedlings at the two–leaf stage with uniform growth characteristics were carefully transplanted into their respective treatment groups. The experimental setup was maintained in a controlled–environment phytotron under the following conditions: constant temperature of 25 ± 1°C, 12 h photoperiod with light intensity of 5,000 lx, and relative humidity of 60–70%. Plants were irrigated twice daily with sterile distilled water. After a cultivation period of 7 d, plants were harvested for morphological analysis, including measurements of shoot length, root length, fresh biomass, and dry biomass. To ensure statistical reliability, each treatment was replicated ten times in a completely randomized design.

To evaluate the soil degradation capability of strain F3Y, we conducted experiments using artificially contaminated soil spiked with oxyfluorfen to a final concentration of 50 mg/kg (dry weight). The inoculation density of F3Y was maintained consistent with previous experimental conditions. The experimental design comprised four distinct treatments: (1) autoclaving–steriled soil (CK1); (2) unsteriled soil (CK2); (3) autoclaving–steriled soil with F3Y (T1); and (4) unsterilized soil with F3Y (T2). All treatments were prepared in triplicate and incubated under controlled dark conditions at 25°C with 60–70% relative humidity for 30 days. At five–day intervals, soil samples (5 g) were collected from each treatment for oxyfluorfen quantification. Herbicide concentrations were determined by HPLC following the validated method of Calderon et al. (2016), which demonstrated excellent recovery efficiency (97%) for oxyfluorfen extraction from soil matrices.

### 2.5 Identification of oxyfluorfen degradation metabolites by strain F3Y

To identify the metabolites of oxyfluorfen degradation, strain F3Y with an initial optical density at 600 nm (OD_600nm_) of 2.0 was inoculated into 60 mL of YPM containing 100 mg/L oxyfluorfen. The inoculated mixture was incubated at 150 rpm and 30°C. Culture filtrates were collected at intervals of 2 h. The extraction of residual oxyfluorfen from the culture medium followed the same method as described in the previous section. After extraction, the dichloromethane solvent was evaporated using a stream of nitrogen at room temperature. Subsequently, an equal volume of methanol was added to redissolve the remaining residues. For the upper aqueous–phase sample, it was centrifuged to remove bacteria. Both the processed samples, the organic–phase sample (after redissolving in methanol) and the centrifuged aqueous–phase sample, were separately filtered through 0.22 μm cellulose acetate and polyamide microporous membranes. The metabolites were identified via ultra-performance liquid chromatography quadrupole–time–of–flight mass spectrometry (UPLC/Q–TOF MS) (Waters Xevo G2–S QTOF, Waters Corporation, Milford, MA, USA). The UPLC/Q–TOF MS instrument was equipped with an Acquity UPLC binary solvent manager, a PDA detector, an Acquity UPLC sample manager FIN coupled to a quadrupole TOF MS, a Xevo G2–S QTOF, and an Acquity UPLC BEH C18 column (2.1 × 100 mm 1.7 μm particle size) (Waters Xevo G2–S QTOF, Waters Corporation, Milford, MA, USA) maintained at 45°C. The mobile phase consisted of 0.1% formic acid–water (A) and 0.1% formic acid-acetonitrile (B). The products were analyzed with gradient program: 0–3 min, from 5 to 20% B; 3–10 min, from 20 to 100% B; 10–12 min, 100% B; and 12–15 min, from 100 to 5% B. The flow rate was 0.4 mL/min, and the injection volume was 1.0 μL. The metabolites were ionized on positive polarity, and the scan range was 50–2000 m/z. All the data were collected and processed via MassLynx V4.1 software and Waters UNIFI software, respectively (Waters Corporation).

### 2.6 Genomic DNA sequencing, assembly, annotation and comparative analysis

The draft genome sequencing of strain F3Y was performed by Shanghai Biozeron Biotechnology Co., Ltd. (Shanghai, China). Genomic DNA was extracted from the collected cells of logarithmic growth using Invitrogen PureLink® Genomic DNA kit, following the manufacturer’s instructions. The quantity and quality of harvested DNA were tested by the NanoDrop ND–1000 Spectrophotometer (Thermo Fisher Scientific). The DNA was purified further using the Quick–DNA Miniprep Plus kit. High qualified DNA sample (OD_260/280_ = 1.8 ~ 2.0, >6 μg) was utilized to construct fragment library. Then, the library was sequenced using Illumina Hiseq×10 platform, and the pair–end reads were assembled by ABySS (Version 2.2.0). GeneMarkS (Version 4.17) was used for coding sequence (CDS) prediction. The predicted CDSs were translated and used to search against the NR (Non–Redundant Protein Sequence), KEGG (Kyoto Encyclopedia of Genes and Genomes), COG (Clusters of Orthologous Groups), and Swiss–Prot databases. The annotated draft genome has been submitted to GenBank with an accession JBLYZF000000000, and the version described in this paper is JBLYZF010000000.

To investigate the conservation and distribution of the *pao* gene cluster, genomes of *S. wittichii* RW1 (oxyfluorfen degrader, GenBank accession numbers: CP000699.1), *M. luteus* NCTC 2665 (a non–degrading congener of F3Y, GenBank accession numbers: LS483396.1), and *Escherichia coli* K12 (a distantly related non–degrading strain, GenBank accession numbers: U00096.3) were retrieved from GenBank. OrthoFinder v2.5.4 (Emms and Kelly, 2019) was used to identify orthologous gene clusters among the four genomes. The *pao* gene cluster in F3Y was used as a query to search for homologous sequences in other strains. Mauve v2.4.0 (Darling et al., 2004) was employed to perform multiple genome alignments and visualize synteny (gene order and orientation) of the *pao* locus.

## 3 Results

### 3.1 Isolation and characterization of the oxyfluorfen–degrading strain

**Figure 1:**
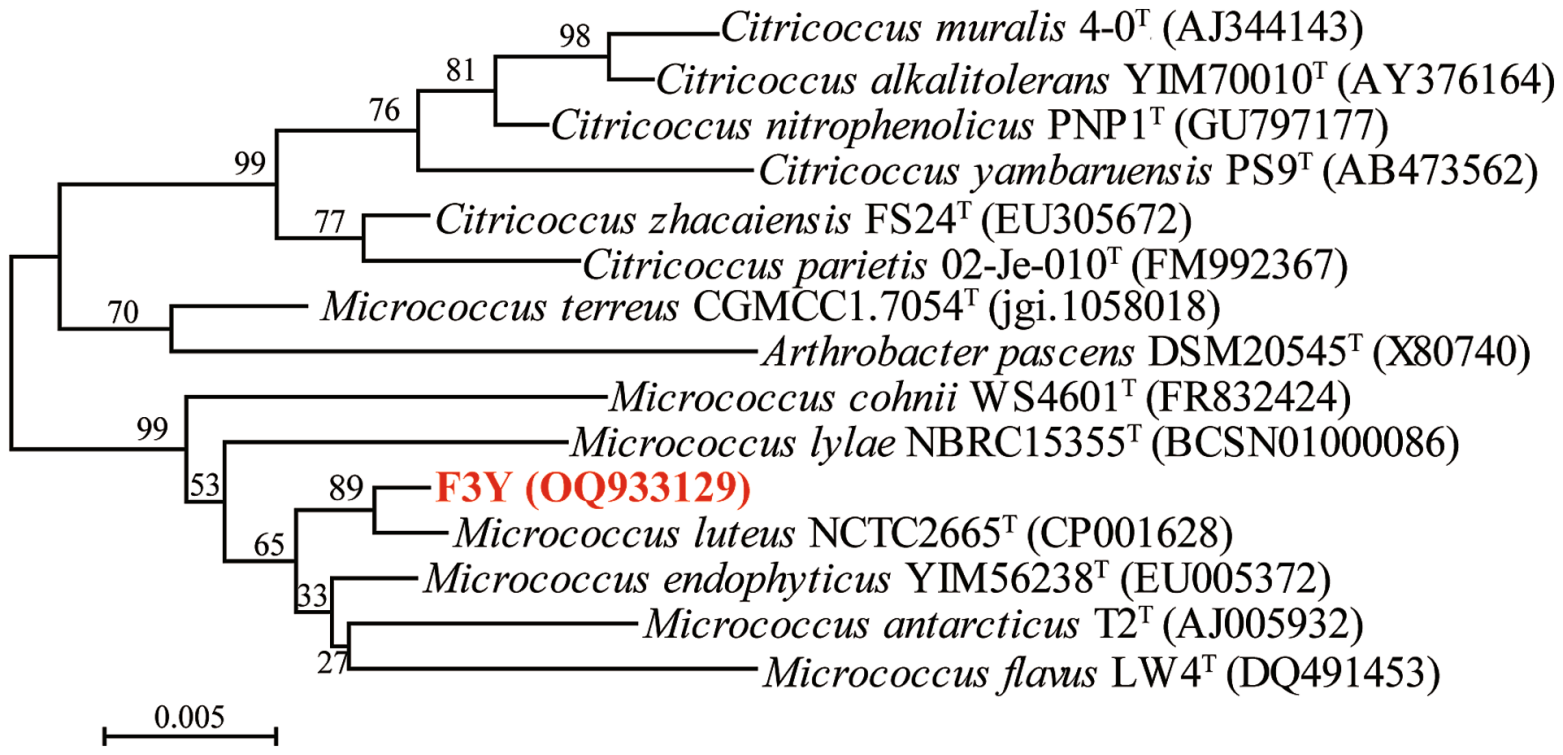
Phylogenetic tree based on the 16S rRNA of strain F3Y and related species. The tree was constructed via the neighbor–joining method. Bootstrap values (expressed as percentages of 1, 000 replications) above 50% are shown at the nodes of branches. Bar, 0.005 represents substitution per nucleotide position.

With multiple rounds of selection and purification, one strain, designated as F3Y, which could degrade oxyfluorfen, was isolated and chosen for further study. Colonies of F3Y grown on LB agar for 2 d were opaque yellow, small, smooth, circular, and convex with entire margins (Supplementary Figure S1A). The cells of the strain were gram–positive, coccus shaped, and nonflagellar, with diameters ranging from 0.53 μm to 0.92 μm (Supplementary Figure S1B). Antibiotic tests indicated that strain F3Y was sensitive to gentamicin, ceftriaxone, and neomycin, but resistant to kanamycin, penicillin, tetracycline, and other antibiotics (Supplementary Table S1). Multiple sequence alignments revealed that strain F3Y was most related to *M. luteus* NCTC 2665^T^ (99.44% similarity). In the neighbor–joining phylogenetic tree (Figure 1), strain F3Y grouped among *Micrococcus* species and formed a subclade with *M. luteus* NCTC 2665^T^. Thus, based on morphological, physiological, and biochemical properties and 16S rRNA phylogenetic analysis, strain F3Y was tentatively identified as *Micrococcus* sp.

### 3.2 Growth of *Micrococcus* sp. F3Y and kinetics of oxyfluorfen degradation

**Figure 2:**
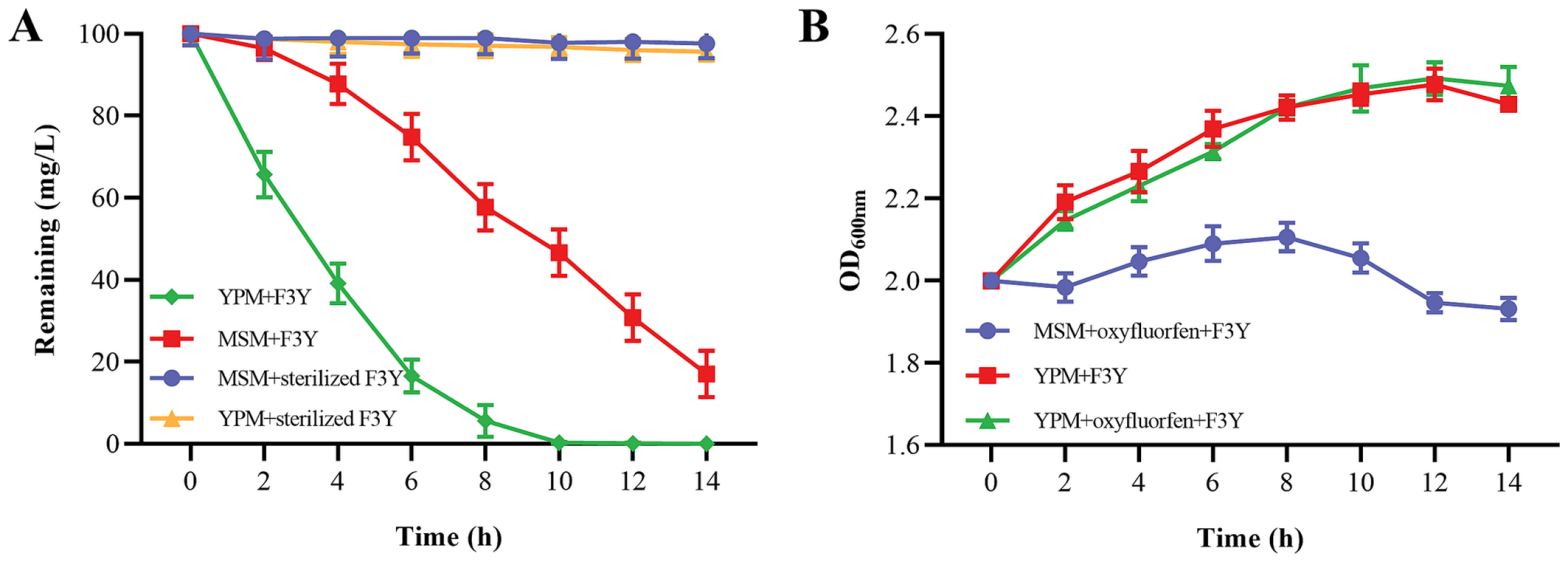
Degradation of oxyfluorfen in MSM and yeast powder–supplimented MSM (YPM) by *Micrococcus* sp. F3Y **(A)** and the growth of the bacterium **(B)**. Error bar indicates the standard deviations of three replicate experiments.

As the degradation process advanced, strain F3Y could degrade 60.87% of the initial 100 mg/L oxyfluorfen within 4 h and completely metabolize 100 mg/L oxyfluorfen within 12 h in YPM (Figure 2A). Moreover, the cell concentration of strain F3Y in YPM gradually increased during this period (Figure 2B). Similar results were observed in MSM, however, the degradation rate in MSM was significantly slower than that in YPM. At 12 h, the residual oxyfluorfen concentration in MSM was detected as 30.79 mg/L, and by 14 h, there was still 17.03 mg/L being detected (Figure 2). Besides, both oxyfluorfen and yeast powder were found to support bacterial growth, with strain F3Y growing faster in YPM compared to MSM supplemented with oxyfluorfen (Figure 2B). These findings suggested that strain F3Y could exhibit high efficiency in degrading oxyfluorfen and was capable of utilizing oxyfluorfen as a sole carbon and energy source for growth.

The effects of different environmental factors on the degradation of oxyfluorfen by strain F3Y were illustrated in Supplementary Figure S2. The degradation rate of 100 mg/L oxyfluorfen within 8 h gradually declined as the pH increased from 7.5 to 9.0 or decreased from 6.5 to 5.0. The optimal pH range for oxyfluorfen degradation was determined to be 7.0–7.5 (Supplementary Figure S2A). Temperature also significantly influenced the degradation capability of strain F3Y, with the optimal temperature identified as 30°C (Supplementary Figure S2B). When the temperature exceeded 37°C, the degradation ability was markedly inhibited, and it was almost entirely lost at 50°C. The impact of the initial oxyfluorfen concentration on biodegradation efficiency was shown in Supplementary Figure S2C. The herbicide was completely degraded at initial concentrations below 100 mg/L. However, when the initial concentration was increased to 150 mg/L, the removal rate dropped to 73.96%. Biodegradation was significantly inhibited at higher oxyfluorfen concentrations (≥200 mg/L), likely due to strain F3Y’s inability to fully detoxify and degrade oxyfluorfen at such levels. As a result, the toxic effects of oxyfluorfen on F3Y cells were not alleviated, leading to a reduction in the degradation rate. The influence of initial biomass on degradation efficiency was presented in Supplementary Figure S2D. As the initial biomass (OD_600nm_) of strain F3Y increased from 0.5 to 3.0, the residual oxyfluorfen concentrations were gradually decreased. However, further increases in biomass (e.g., to 2.5 and 3.0) did not enhance the degradation efficiency significantly. Therefore, the optimal initial biomass for strain F3Y in this study was determined to be OD_600nm_ = 2.0.

### 3.3 The remediation of oxyfluorfen–contaminated soil by strain F3Y

**Figure 3:**
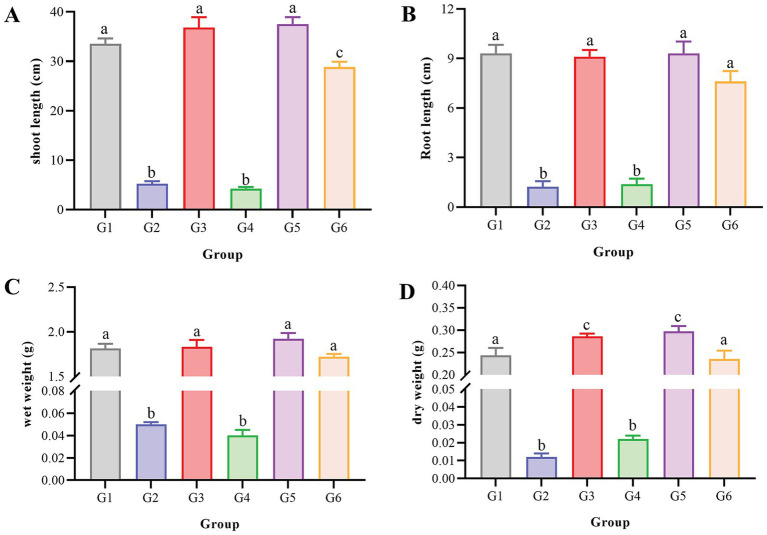
Morphological parameters of oxyfluorfen–sensitive soybean (*Glycine max*) plants after 7 d of growth under different treatments. **(A)**, shoot length; **(B)**, root length; **(C)**, fresh biomass; **(D)**, dry biomass. G1, −oxyfluorfen, –F3Y; G2, +oxyfluorfen, –F3Y; G3, −oxyfluorfen, +inactivated F3Y; G4, +oxyfluorfen, +inactivated F3Y; G5, −oxyfluorfen, +F3Y; and G6, +oxyfluorfen, +F3Y. Different letters (a, b, and c) denote significant effects of different treatments.

The pot experiment utilized soybean (*Glycine max*) as a bioindicator to assess the detoxification capacity of strain F3Y in oxyfluorfen–contaminated soil. Comprehensive growth parameters were quantitatively analyzed across all treatment groups (Figure 3, Supplementary Figure S3). Control groups without oxyfluorfen (Groups 1, 3, 5) showed normal soybean development, with shoot lengths of 33.5 cm, 36.8 cm, and 37.5 cm, respectively, (Figure 3A), confirming the non–phytotoxic nature of strain F3Y. In stark contrast, oxyfluorfen–exposed groups without active F3Y (Groups 2, 4) exhibited severe phytotoxicity, manifesting as dramatically stunted growth (shoot lengths: 5.21 cm and 4.22 cm) and complete mortality in some specimens (Supplementary Figure S3). Remarkably, Group 6 (oxyfluorfen+active F3Y) demonstrated near–complete recovery, with shoot lengths reaching 28.8 cm, representing an 82% restoration compared to healthy controls. This recovery pattern was consistently observed across all measured parameters: root length (Figure 3B), fresh biomass (Figure 3C), and dry biomass (Figure 3D).

Soil degradation kinetics revealed striking differences between treatments (Supplementary Figure S4). After 30 days, sterile control soil (CK1) showed minimal degradation (4.94%), while native microflora in unsterilized soil (CK2) achieved 40.94% degradation. Introduction of strain F3Y dramatically enhanced degradation: in sterile soil (T1), 28.7% degradation occurred within 5 days, progressing to complete elimination by day 30. Most notably, in unsterilized soil (T2), degradation accelerated to 47.7% by day 5, with near–total herbicide removal (98.2%) by day 25, suggesting synergistic interactions between F3Y and indigenous microbiota.

### 3.4 Identification of the metabolites in the degradation of oxyfluorfen by strain F3Y

Samples were collected at regular intervals during the oxyfluorfen degradation process by strain F3Y, and the oxyfluorfen content in these samples was quantified using HPLC (Supplementary Figure S5). The oxyfluorfen concentration continued to decrease until it was fully degraded by 12 h (Supplementary Figure S5G). In the organic phase, M1 (retention time: 3.64 min) was first detected at 2 h, and its concentration continued to increase in the first 4 h, but a decrease in its content was observed at 6 h; while M2 (4.91 min) was also detected at 2 h, and its content continued to decrease subsequently until it could no longer be detected at 8 h (Supplementary Figures S5B–E). In the aqueous phase, the transformation of intermediate products was more complex. Four products, M3 (3.11 min), M4 (4.77 min), M5 (7.80 min), and M6 (8.91 min) were all first detected at 2 h. But the former two products disappeared at 4 h; the concentration of the third product experienced a period of increase, and then it was found to have decreased at 6 h, until it completely gone at 8 h; and the content of M6 gradually decreased until it vanished at 8 h (Supplementary Figures S5B–E). M7 (3.99 min) was first detected at 4 h, and it could not be detected at the next sampling time point; M8 (3.69 min), first detected at 6 h, was fully metabolized by strain F3Y within 4 h (Supplementary Figures S5C–F). M9 (6.68 min) and M10 (8.31 min), observed at 10 and 12 h, respectively, were the products that appeared the latest, neither of them was detected at the next sampling time point (Supplementary Figures S5F–H). In a word, all the metabolites were finally gone, indicating that they could all be further utilized and completely degraded by strain F3Y.

UPLC/Q–TOF MS was utilized to identify the degradation products of oxyfluorfen (Supplementary Table S2 and Supplementary Figure S6). The parent compound, oxyfluorfen (m/z = 361.01), was detected with a protonated molecular ion at m/z 362.03 [M + H]^+^ (Supplementary Figure S6A). The intermediate compounds were characterized based on their exact mass and characteristic fragment ion peaks as follows: *N*-acetylaminooxyfluorfen (M1, m/z = 373.07, [M + H]^+^) (Supplementary Figure S6B), aminooxyfluorfen (M2, m/z = 331.06, [M + H]^+^) (Supplementary Figure S6C), 3–ethoxy–4–nitrophenol (M3, m/z = 183.05, [M + H]^+^) (Supplementary Figure S6D), 4–ethoxy–5–nitrobenzene-1,2–diol (M4, m/z = 199.05, [M + NH_4_]^+^) (Supplementary Figure S6E), *N*–(2–ethoxy–4, 5–dihydroxyphenyl)acetamide (M5, m/z = 211.08, [M + NH_4_]^+^) (Supplementary Figure S6F), *N*–(2–ethoxy–4–hydroxyphenyl)acetamide (M6, m/z = 195.09, [M + NH_4_]^+^) (Supplementary Figure S6G), 4–amino–3–ethoxyphenol (M7, m/z = 153.08, [M + NH_4_]^+^) (Supplementary Figure S6H), (2–ethoxy–4, 5–dihydroxyphenyl)carbamic acid (M8, m/z = 213.06, [M + NH_4_]^+^) (Supplementary Figure S6I), ((*2E, 4E*)–4–ethoxy–1, 6–dihydroxyhexa–2,4–dien–3–yl)carbamic acid (M9, m/z = 217.10, [M + Na]^+^) (Supplementary Figure S6J), (*2E,4E*)–3–acetamido–4–ethoxyhexa–2,4–dienedioic acid (M10, m/z = 243.07, [M + NH_4_]^+^) (Supplementary Figure S6K), and (*2E,4E*)–2–chloro–4–(trifluoromethyl)hexa–2,4-dienedioic acid (M11, m/z = 243.98, [M + H]^+^) (Supplementary Figure S6L).

**Figure 4:**
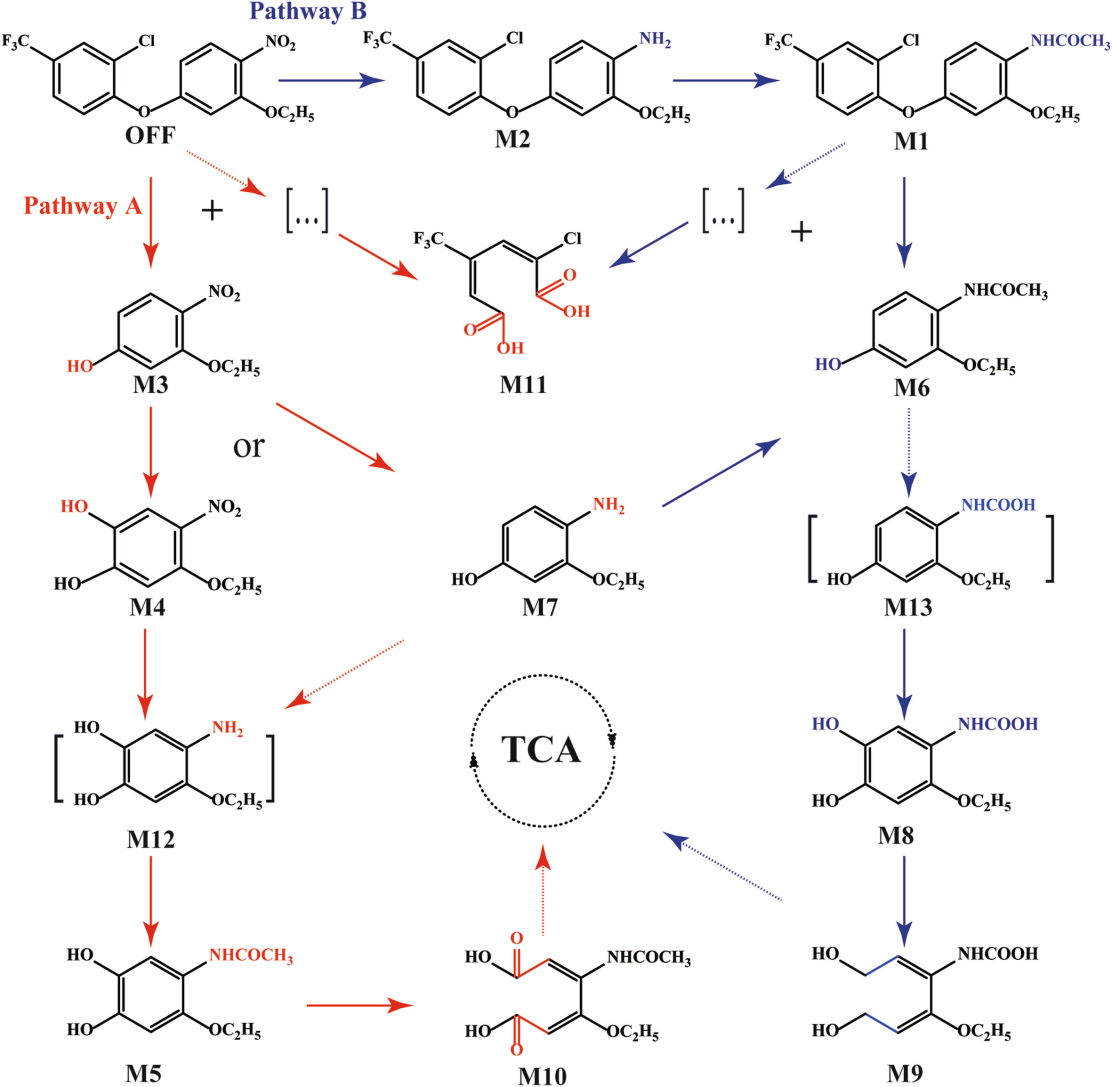
Proposed metabolic pathway of oxyfluorfen degradation by strain F3Y.

Based on these findings, a comprehensive degradation pathway for oxyfluorfen involving two distinct initial metabolic patterns in strain F3Y was proposed (Figure 4). Pathway A began with the direct cleavage of the diaryl ether bond, resulting in the immediate formation of M3. This intermediate might undergo either hydroxyl oxidation to produce M4 or nitro reduction to yield M7. Both M4 and M7 were subsequently converted through complementary reactions (nitro reduction and hydroxyl oxidation, respectively) to form the presumed intermediate 4–amino–5–ethoxybenzene–1,2–diol (M12). Following *N*–acetylation, M12 was transformed into M5, which underwent aromatic ring cleavage to produce M10. Pathway B initiated with the nitro reduction of oxyfluorfen, followed by *N*–acetylation to form metabolite M1. The subsequent hydrolysis of the diaryl ether bond in M1 generated M6, which may undergo *O*–demethylation to produce (2–ethoxy–4–hydroxyphenyl)acetamide carbamic acid (M13) (not detected in this study). M13 was then transformed into M8 through hydroxyl oxidation, followed by aromatic ring cleavage to yield the final product M9. The terminal products from both pathways were ultimately assimilated into the tricarboxylic acid (TCA) cycle. Additionally, M11 might be derived from an intermediate product generated during the cleavage of the oxyfluorfen and M1 ether bond, which was not detected due to its low concentration or instability in the HPLC analysis.

### 3.5 Genes related to oxyfluorfen metabolism

**Figure 5:**
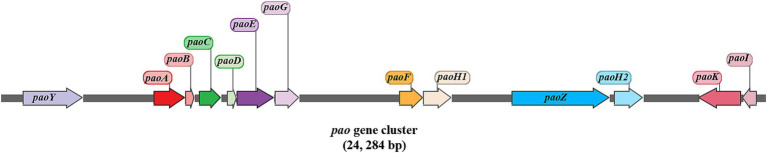
Genetic map of the hypothetical function of the different proteins.

The draft genome of strain F3Y was sequenced to identify potential genes associated with oxyfluorfen metabolism. The genome assembly revealed a total size of 2,485,282 base pairs (bp) with a G + C content of 73.44%. Sequence assembly resulted in 148 contigs, with an N50 length of 50,633 bp. Gene prediction identified 2,166 protein–coding sequences (CDSs) in the draft genome. Functional annotation of these CDSs was performed through comprehensive homology analysis against multiple databases, including NR, KEGG, COG, and Swiss–Prot. Notably, genes potentially involved in oxyfluorfen degradation and intermediate metabolism were supposed and summarized in Supplementary Table S3. In the draft genome scaffold of strain F3Y, a 24,284 bp gene cluster named *pao* was proposed. This cluster consisted of thirteen genes, namely *paoY* and *paoABCDEFGH1H2IKZ* (as shown in Figure 5). Among these genes, *paoY* was determined to encode phenol–2–monooxygenase, an enzyme known to catalyze the hydroxylation of phenol to catechol. Given the structural similarity between phenol and several identified metabolites (M3, M6, M7, and M13), we hypothesized that *paoY* may similarly hydroxylate these phenolic intermediates during oxyfluorfen degradation. The remaining twelve genes appeared to constitute a complete phenylacetic acid (PhAc) degradation pathway, organized into four functional modules: (*i*) PhAc activation, *paoK* encoded a phenylacetyl–CoA ligase (PhAc–CoAL), which convert PhAc to PhAc–CoA; (*ii*) ring–hydroxylation, *paoABCDE* encoded the five subunits of phenylacetyl–CoA epoxidase, forming a multicomponent complex for PhAc–CoA hydroxylation; (*iii*) ring cleavage, *paoZ* encoded a bifunctional ring–opening enzyme; and (*iv*) *β*–oxidation–like system, *paoFGHI* encoded enzymes homologous to fatty acid *β*–oxidation components (enoyl–CoA hydratase, isomerase, dehydrogenase, and thioesterase). Comparative genomic analysis revealed striking conservation of this cluster across diverse bacteria. The twelve core genes (*paoYABCDEFGH1IKZ*) maintained perfect synteny in all tested strains, including the oxyfluorfen–degrader *S. wittichii* RW1, the non–degrader *M. luteus* NCTC 2665 (a congener), and the phylogenetically distant *E. coli* K12. Notably, *paoH2* showed restricted distribution, being present only in *M. luteus* NCTC 2665 (Supplementary Figure S7).

## 4 Discussion

In this study, strain F3Y was isolated and identified as the genus *Micrococcus*, which was part of the phylum *Actinomycetota*. While bacteria within this phylum were primarily recognized for their ability to synthesize bioactive compounds, their potential for degrading organic pollutants has received comparatively less attention. Nevertheless, few researches have demonstrated the functional degradation of recalcitrant compounds by *Micrococcus* species. For instance, *M. yunnanensis* strain HY001 was reported to reduce soil paclobutrazol residue by 75.18% over 5 months (Jiang et al., 2025); *M. flavus* RS124 effectively degraded high-density polyethylene (Sardar, 2025); *M. aloeverae* MAGK3 degraded 66.65% of 500 μL/L malathion within 15 d (Dar et al., 2022); and *M.* sp. KS2 achieved 99.67% degradation of 289.19 mg/L dimethyl phthalate under optimal conditions (pH 7.05, 31.5°C) (Patil and Jena, 2023). *Micrococcus* sp. F3Y was demonstrated to utilize oxyfluorfen as a carbon source for growth and completely degrade the herbicide in the present study. Further exploration of the metabolic capabilities of this strain will not only highlight the versatility of *Actinomycetota* but also expand our understanding of their ecological roles, particularly their biodegradation potential, beyond their well-established capacity for secondary metabolite production.

To date, several strains capable of degrading oxyfluorfen have been isolated and characterized. Strain *B licheniformis* ATCC 21415 was used to ferment sewage sludge to prepare two biostimulants BS1 and BS2, which were used and found decrease the concentration of oxyfluorfen by 37.5 and 25%, respectively, after 72 d (Tejada et al., 2022). *B methylotrophicus* A1 and *Streptomyces* sp. B1 demonstrated 450 mg/L oxyfluorfen degradation efficiencies of 81.33 and 79.75%, respectively, over 15 d in minimal media (Bhatt, 2017). *A. chroococcum* (Beijerinck) demonstrated the ability to degrade 60% of 240 mg/L oxyfluorfen within 7 d (Chakraborty et al., 2002), and *S. wittichii* RW1 degraded approximately 75% of 100 mg/L oxyfluorfen within 7 d in M9 medium (Keum et al., 2008). Additionally, *C. aquifrigidense* R21 achieved a degradation rate of 92.1% for 50 mg/L oxyfluorfen within 5 d (Zhao et al., 2016). Other reported strains exhibited degradation efficiencies of on higher than 95.6% for 45 mg/L oxyfluorfen within 21 d (Mohamed et al., 2011). Although these strains possessed the ability to degrade oxyfluorfen at relatively high concentrations, their degradation rates and extents were markedly lower compared to those of F3Y. Under optimized conditions, F3Y could completely mineralize 100 mg/L of oxyfluorfen within 12 h. Furthermore, the strain maintained robust degradation efficiency across various environmental conditions, removing over 62% of 100 mg/L oxyfluorfen within a pH range of 6.5–8.0, and retaining more than 55% degradation efficiency at temperatures between 25–42°C. Additionally, F3Y achieved a degradation rate exceeding 74% when the initial oxyfluorfen concentration was ≤150 mg/L. This striking difference highlighted the exceptional catalytic activity and adaptability of F3Y. Morever, F3Y exhibited remarkable soil remediation efficiency, alleviating oxyfluorfen–induced phytotoxicity in soybean (shoot length recovery to 82% of controls) and degrading 98.2% of the herbicide in unsterilized soil, which significantly surpassing the degradation by native microbiota (40.94%). This finding suggested synergistic interactions between F3Y and indigenous microbes (the impact data of strain F3Y on soil microbial community was not displayed), highlighting its practical applicability in agricultural soil remediation.

Here, the intermediate and terminal products were identified so as to elucidate the complete degradation pathways of oxyfluorfen by strain F3Y. The results revealed that strain F3Y primarily degraded oxyfluorfen through two distinct initial modes, initiated by either nitro reduction or diaryl ether cleavage. Although these initial reactions align with those reported in other oxyfluorfen–degrading bacteria (Zhao et al., 2016; Keum et al., 2008; Chakraborty et al., 2002), the subsequent reactions and the extent of degradation differ significantly, highlighting the unique metabolic capabilities of strain F3Y. In the first pathway, M3, generated through direct diaryl ether cleavage of oxyfluorfen, underwent a series of transformations, including monooxygenation, nitro reduction, and *N*–dealkylation, to form M4 and M5. These two compounds, identified as novel intermediates in oxyfluorfen degradation, were detected for the first time in this study. Subsequently, M5 was converted into M10 through ring-opening reactions, ultimately entering the tricarboxylic acid (TCA) cycle for complete mineralization. However, *A. chroococcum* (Chakraborty et al., 2002) and *C. aquifrigidense* R21 (Zhao et al., 2016) convert M3 into 4–nitrobenzene–1,3–diol via *O*–dealkylation, with no further transformation of this compound. In the second pathway, oxyfluorfen initially degraded by nitroreduction, and then underwent *N*–acetylation and ether bond cleavage to generate M6. Strain F3Y could further converte M6 into a newly identified intermediate, M8, through *O*–demethylation and monooxygenation. M8 was then transformed into the final product M9, which entered the TCA cycle, achieving complete mineralization. In *A. chroococcum* and *C. aquifrigidense* R21, the same intermediate M6 was also iedtified, but the two strain did not have the ability to further utilize and degrade M6. Notably, strain F3Y also exhibited the ability to degrade chlorinated organic compounds, as evidenced by the formation of M11 through the opening of the chlorobenzene ring in the oxyfluorfen structure. This capability highlighted the strain’s potential to effectively mitigate the toxicity of oxyfluorfen to sensitive plants during bioremediation processes, particularly in environments contaminated with chlorinated pollutants. In conclusion, this study provided a comprehensive understanding of the metabolic fate of oxyfluorfen in strain F3Y. We detected a greater number of intermediate compounds compared to previous studies, and both metabolic pathways demonstrated the ring-opening degradation processes and ultimately lead to its complete mineralization. These findings underscore the complex and efficient degradation mechanisms employed by strain F3Y, offering valuable insights for the bioremediation of oxyfluorfen–contaminated environments.

Although the *pao* gene cluster was initially hypothesized to participate in oxyfluorfen degradation, a closer examination revealed a more nuanced role. The widespread presence of the *pao* core genes in both oxyfluorfen–degrading strains like *S. wittichii* RW1 and non–degrading strains such as *M. luteus* NCTC 2665 and *E. coli* K12, along with their conserved synteny, strongly suggested that this cluster primarily functions in the metabolism of aromatic compounds. This was a function that has been conserved across a diverse range of bacterial lineages. To illustrate this point, consider the *paa* operon, which has been identified in *E. coli* W (Grishin et al., 2011; Fernández et al., 2006; Ferrández et al., 1998) and *Acinetobacter baumannii* (Hooppaw et al., 2022). Similarly, the *pha* operon has been reported in *Pseudomonas putida* U (Olivera et al., 1998). These operons, which were homologous to *pao*, endowed bacteria with the ability to utilize a wide array of aromatic compounds as sources of carbon and energy. This included substances like phenylacetic acid (Sathyanarayanan et al., 2019; Teufel et al., 2010), 2–phenylethylamine, phenylacetaldehyde, trans–styrylacetic acid, styrene, and various phenylalkanoic acids with an even number of carbon atoms (Grishin et al., 2011). In the case of strain F3Y, the *pao* cluster might play a part in the ring–cleavage of oxyfluorfen intermediates, such as M5 and M8. However, its presence in non–degrading strains clearly indicated that it alone was insufficient for oxyfluorfen degradation. Instead, the unique ability to degrade oxyfluorfen likely depended on additional enzymes. For example, nitroreductases or ether hydrolases might be required for the initial nitro reduction or diaryl ether cleavage steps. These enzymes could then work in tandem with the *pao* pathway to achieve complete mineralization of oxyfluorfen.

## 5 Conclusion

In this study, an actinobacteria strain F3Y, capable of utilizing oxyfluorfen as the sole carbon source for growth, was isolated from activated sludge and taxonomically identified as *Micrococcus* sp. Under laboratory conditions, strain F3Y exhibited efficient oxyfluorfen degradation, with optimal performance at pH 7.0, 30°C, initial pollutant concentrations ≤150 mg/L, and a cell density of OD_600nm_ = 2.0. Notably, F3Y achieved >98% removal of 50 mg/kg oxyfluorfen in soil within 25 d, concurrently alleviating its phytotoxic effects and restoring soybean growth to near–normal levels. Eleven degradation products were identified, six of which are reported here for the first time. Two distinct initial degradation pathways were proposed, both involving aromatic ring cleavage and completel mineralization of oxyfluorfen. A conserved *pao* gene cluster was proposed, which was hypothesized to participate in the aromatic ring cleavage of degradation intermediates (e.g., M5, M8). However, its presence in both degrading and non–degrading strains suggested that it functions as part of a broader aromatic metabolism network, with strain–specific enzymes likely responsible for the initial steps of oxyfluorfen degradation. These findings enhance our understanding of the biochemical pathways underlying oxyfluorfen degradation and provide a foundation for developing innovative bioremediation strategies.

While laboratory results are promising, the applicability of F3Y for *in situ* bioremediation requires validation in real–world agricultural systems, where environmental conditions differ significantly from controlled settings. The strain’s functional pH range (6.5–8.0) aligns with neutral to slightly alkaline farmland soils (typical pH 5.5–8.0), and its temperature tolerance (25–42°C) partially overlaps with seasonal temperate in temperate to tropical regions (15–35°C), suggesting potential for seasonal deployment during warmer periods (e.g., spring or summer) to leverage its 30°C optimum. Given that oxyfluorfen residues in agricultural soils typically range from 0.002–0.03 mg/kg (well below the 50 mg/kg tested in this study), F3Y is likely effective for remediating conventional contamination levels. However, challenges remain for translating laboratory findings to field applications, including enhancing the strain’s environmental adaptability, persistence, and stability across diverse soil types and climatic conditions. Future research should prioritize these aspects to facilitate the practical implementation of F3Y–based bioremediation technologies.

## Data Availability

The datasets presented in this study can be found in online repositories. The names of the repository/repositories and accession number(s) can be found in the article/Supplementary material.

## Footnotes

^1^ http://www.ezbiocloud.net/
