# Supplementary material for: Metabolism of oxyfluorfen by actinobacteria Micrococcus sp. F3Y
Source: Front Microbiol. 2025 May 9;16:1599015. doi: 10.3389/fmicb.2025.1599015 (PMC12098357; doi:10.3389/fmicb.2025.1599015)
Supplement: Supplementary file 1 [file Supplementary_file_1.zip › Supplementary Figures.DOCX]

**Metabolism of oxyfluorfen by actinobacteria** ***Micrococcus* sp. F3Y**

**Li Yao^1, †^, Yue Wen^1, 2, †^, Yuting Sha^1^, Leqin Wang^1^, Xianrui Bi^1^, Shuhan Si^1^, Min Shen^1^, Shusong Zhang^1^, Haiyan Ni^3, *^**

^1^College of Marine and Bio‒Engineering, Yancheng Teachers University, Yancheng 210095, China

^2^College of Biotechnology and Pharmaceutical Engineering, Nanjing Tech University, Nanjing 211816, China

^3^Nanchang Key Laboratory of Microbial Resources Exploitation & Utilization from Poyang Lake Wetland, College of Life Sciences, Jiangxi Normal University, Nanchang 330022, China

^†^These authors contributed equally to this work.

**^*^Correspondence:**

Haiyan Ni

E-mail address: [nihaiyan16@163.com](mailto:nihaiyan16@163.com).

Supplementary Material


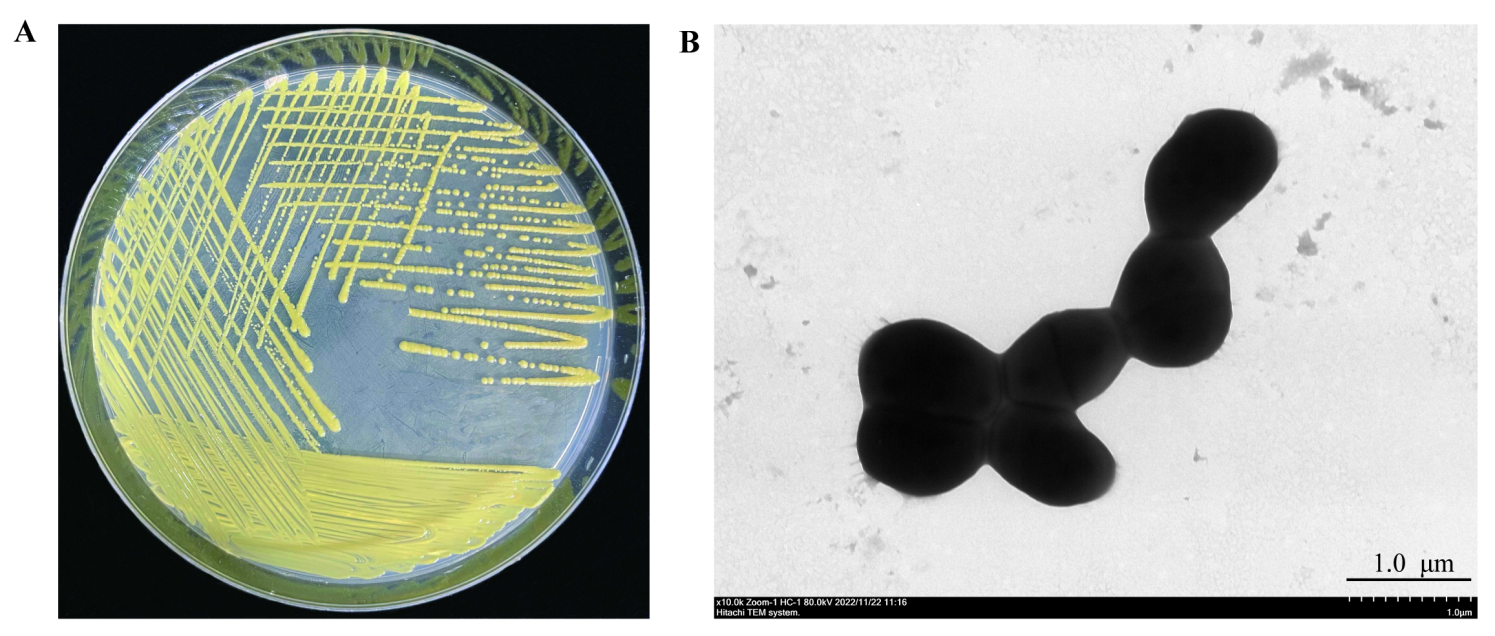


**Supplementary Figure 1.** Colony morphology of strain F3Y on the LB plates (A) and micromorphology of strain F3Y characterized by TEM (B).


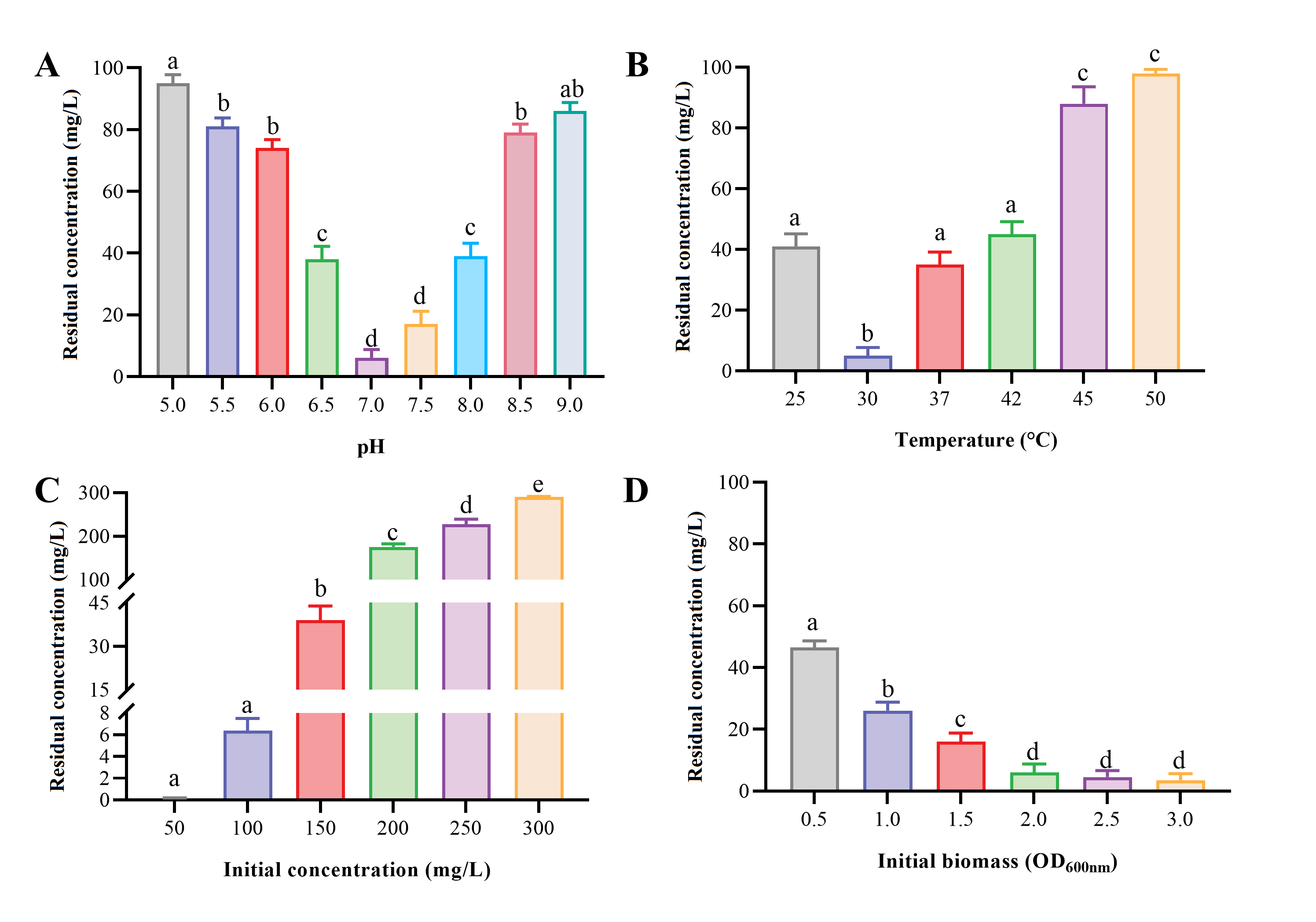


**Supplementary Figure 2.** The oxyfluorfen degradation characteristics of strain F3Y under various environmental conditions. A, pH; B, temperature; C, initial concentration of oxyfluorfen; D, initial biomass (OD_600nm_). The Y‒axis represents the residual concentration of oxyfluorfen. Different letters (a, b, c, d and e) denote significant effects of different treatments.


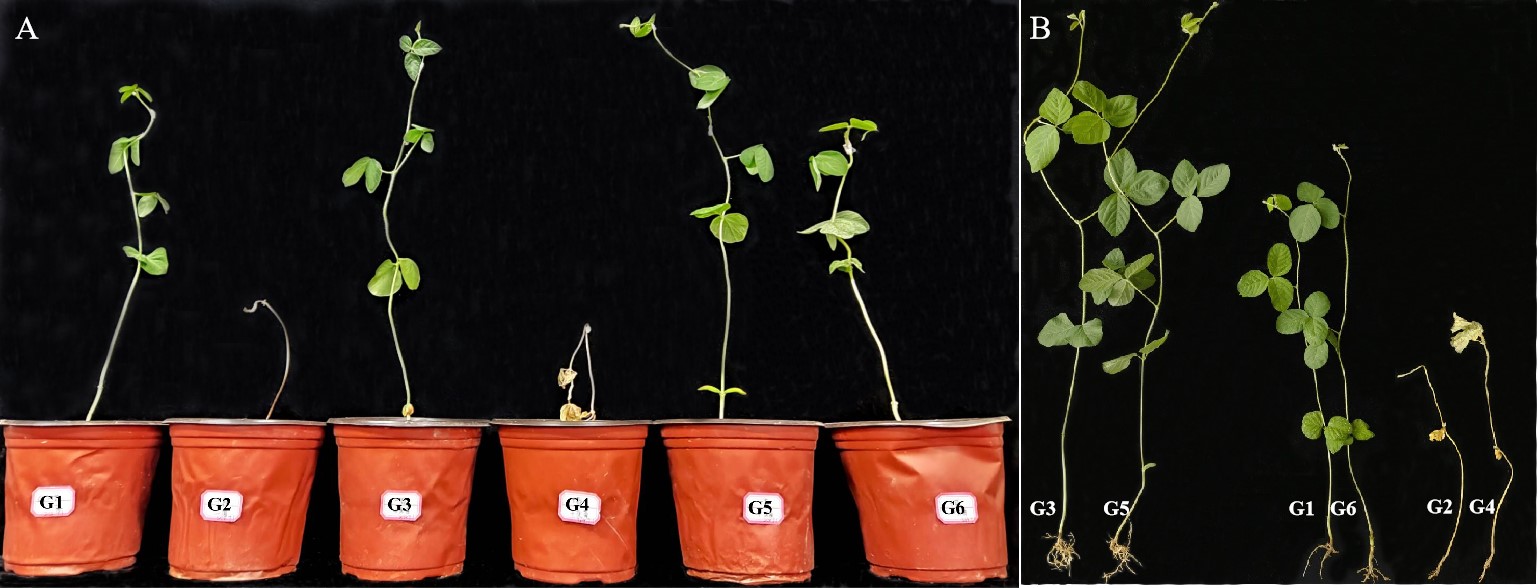


**Supplementary Figure 3.** Effects of different treatments on the morphology of soybean (*Glycine max*) plants after 7 days of cultivation. G1, negative control (‒oxyfluorfen, ‒F3Y), soil without oxyfluorfen and F3Y inoculation; G2, **oxyfluorfen toxicity assessment (+oxyfluorfen,** ‒**F3Y),** soil amended with 0.06 mg/kg oxyfluorfen; G3, **inactivated F3Y impact (**‒**oxyfluorfen, +inactivated F3Y),** soil inoculated with inactivated F3Y strain; G4, **inactivated F3Y in oxyfluorfen**‒**contaminated soil (+oxyfluorfen, +inactivated F3Y),** soil containing both 0.06 mg/kg oxyfluorfen and inactivated F3Y strain;G5, viable F3Y impact (‒oxyfluorfen, +F3Y), oxyfluorfen‒free soil inoculated with viable F3Y strain; and G6, **bioremediation potential assessment (+oxyfluorfen, +F3Y),** soil treated with both 0.06 mg/kg oxyfluorfen and viable F3Y strain. A, schematic representation of potted soybean seedlings; B, whole‒plant schematic diagram of soybean seedlings.


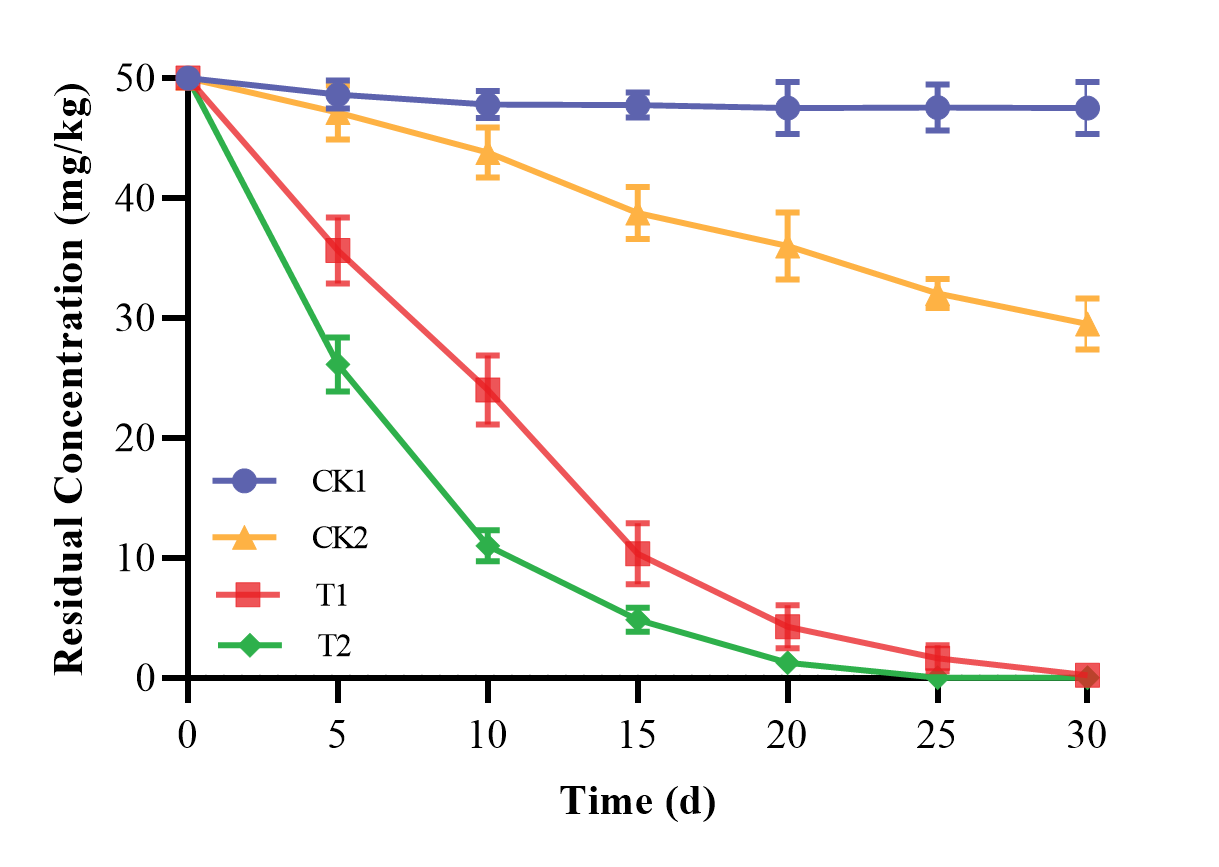


**Supplementary Figure 4.** Degradation of oxyfluorfen in various soils. CK1, sterilized soil; CK2, unsterilized soil; T1, sterilized soil with strain F3Y; T2, unsterilized soil with strain F3Y . The error bars represent the standard errors of three replicates.


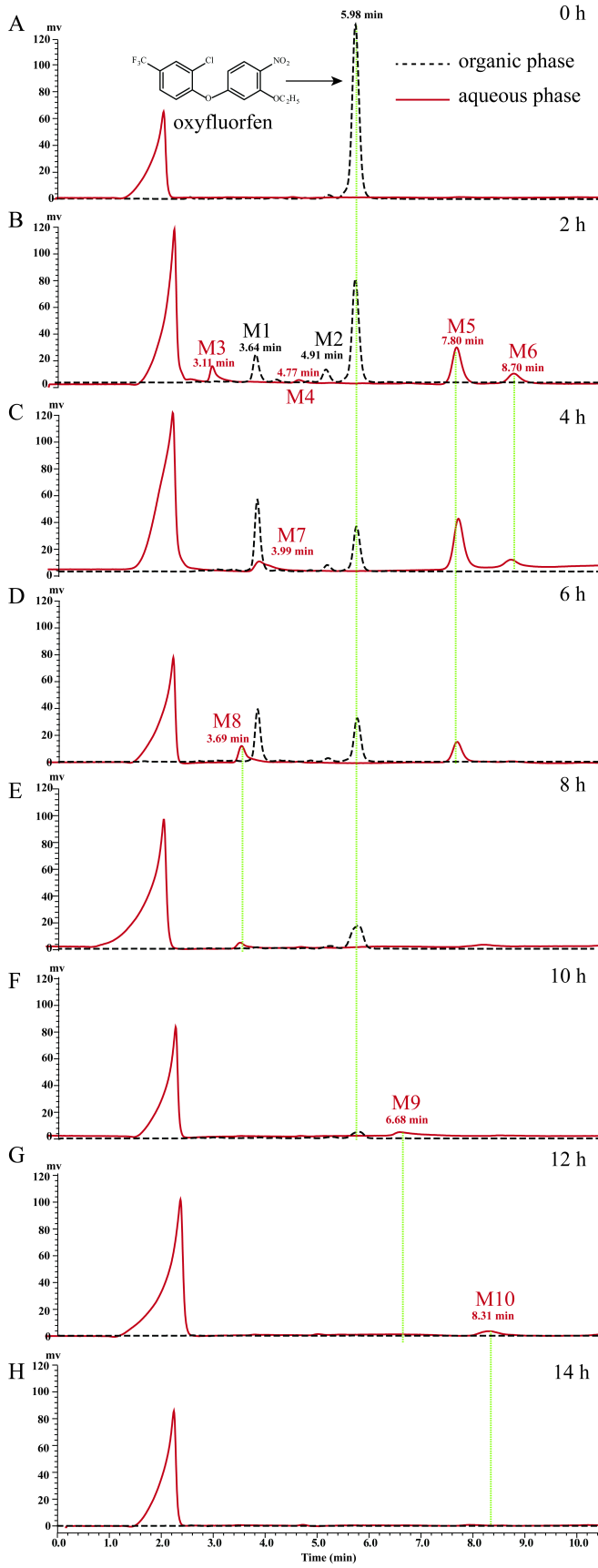


**Supplementary Figure 5.** HPLC chromatograms of compound oxyfluorfen degradation by strain F3Y at A, 0 h; B, 2 h; C, 4 h; D, 6 h; E, 8 h; F, 10 h; G, 12 h; H, 14 h. The dotted line indicates organic phase, and the solid line indicates aqueous phase.


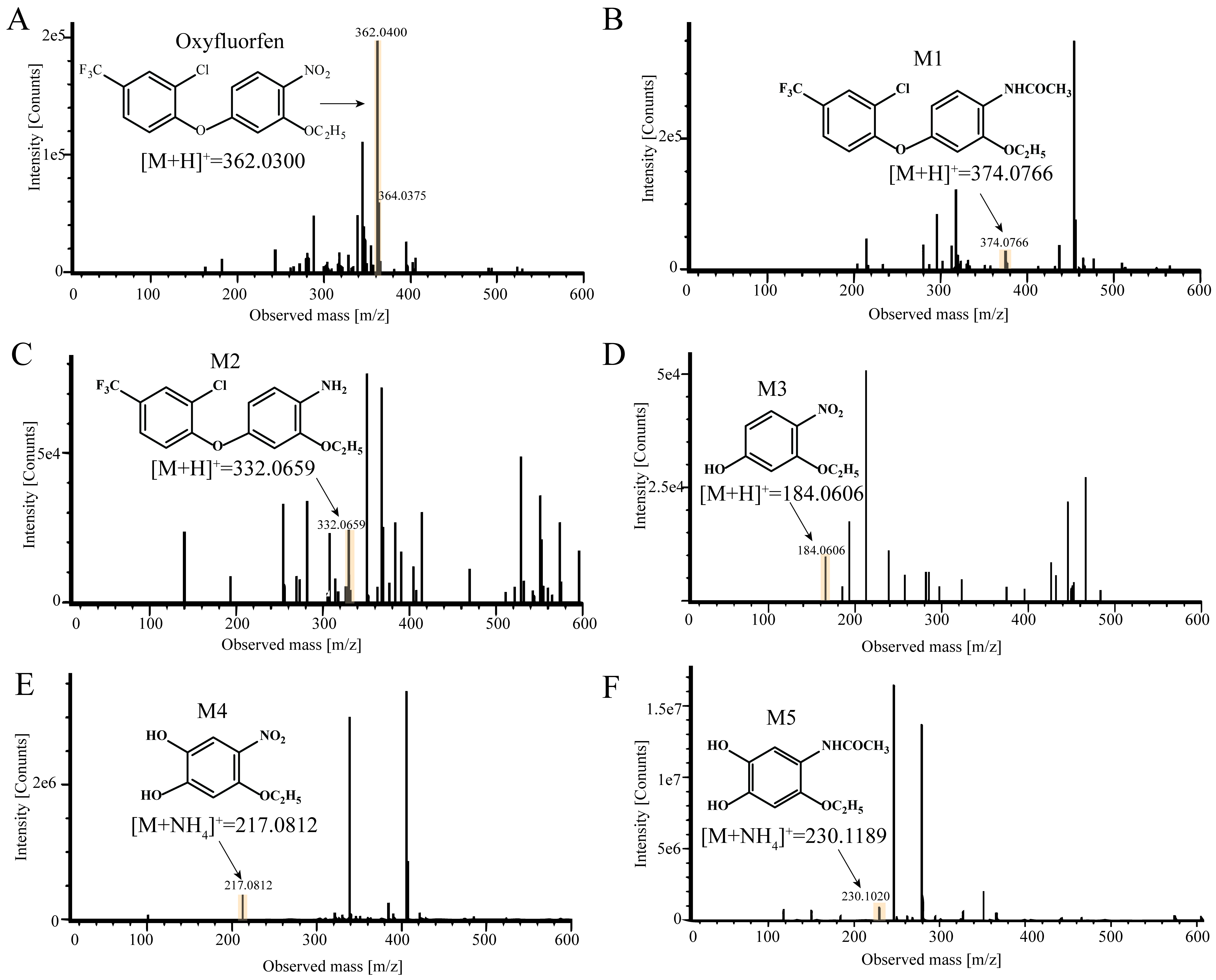


**
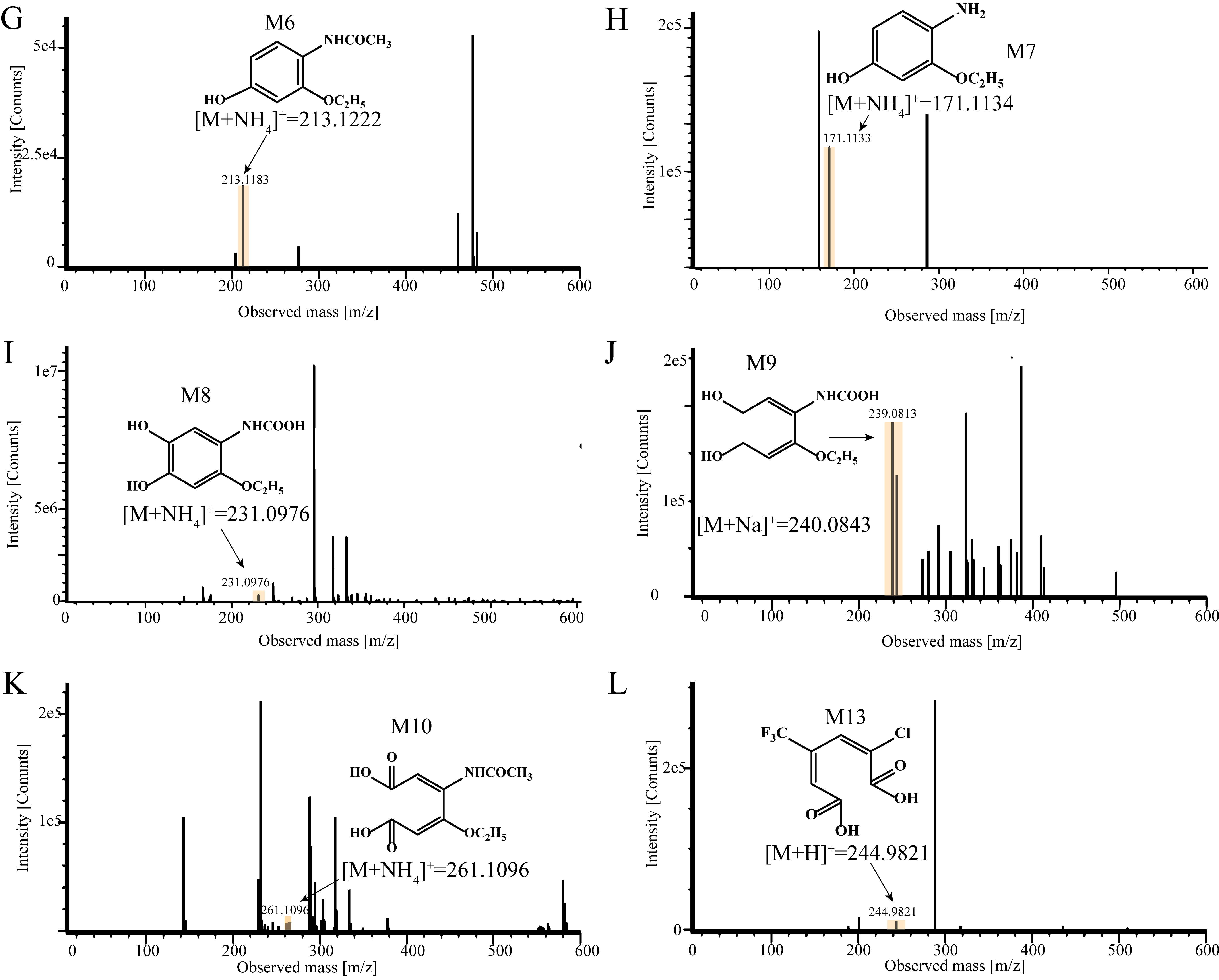
**

**Supplementary Figure 6.** Mass spectra of oxyfluorfen (A) and its metabolites (B‒L) produced by strain F3Y.


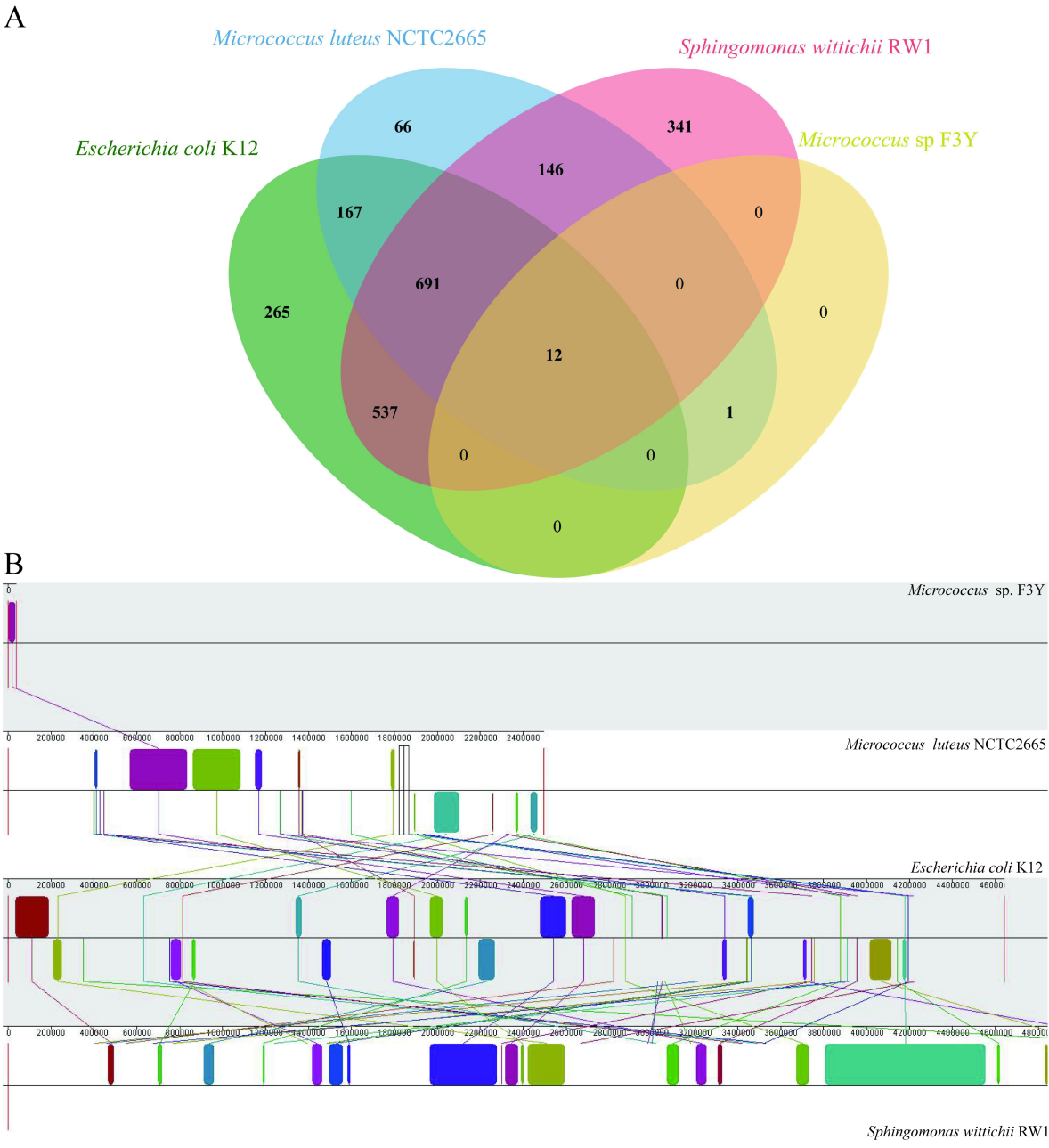


**Supplementary Figure 7.** Comparative genomic analysis of the *pao* gene cluster in *Micrococcus* sp. F3Y and reference strains. **A, Venn diagram of** pao **cluster orthologs.** Shared and unique genes within the pao cluster are illustrated among ***Micrococcus* sp. F3Y (oxyfluorfen**‒**degrading, GenBank accession numbers: JBLYZF010000000), *Sphingomonas wittichii* RW1 (oxyfluorfen**‒**degrading, GenBank accession numbers: CP000699.1), *Micrococcus luteus* NCTC 2665 (non**‒**degrading congener, GenBank accession numbers: LS483396.1), *Escherichia coli* K12 (non**‒**degrading distantly related strain, GenBank accession numbers: U00096.3).** Numbers represent counts of orthologous genes conserved in overlapping regions or unique to specific strains. **B, Synteny analysis of the** pao **cluster. Whole**‒**genome alignment of *pao* cluster regions across the four strains. Colored blocks denote homologous genes, with connecting lines indicating conserved syntenic relationships. Gene orientation (forward/reverse strand) is represented by block direction (up/down). The *pao* cluster (highlighted in purple) exhibits perfect synteny among all strains, emphasizing its evolutionary conservation.**
