# Supplementary material for: Metabolism of oxyfluorfen by actinobacteria Micrococcus sp. F3Y
Source: Front Microbiol. 2025 May 9;16:1599015. doi: 10.3389/fmicb.2025.1599015 (PMC12098357; doi:10.3389/fmicb.2025.1599015)
Supplement: Supplementary file 1 [file Supplementary_file_1.zip › Supplementary Tables.docx]

**Metabolism of oxyfluorfen by actinobacteria** ***Micrococcus* sp. F3Y**

**Li Yao^1, †^, Yue Wen^1, 2, †^, Yuting Sha^1^, Leqin Wang^1^, Xianrui Bi^1^, Shuhan Si^1^, Min Shen^1^, Shusong Zhang^1^, Haiyan Ni^3, *^**

^1^College of Marine and Bio‒Engineering, Yancheng Teachers University, Yancheng 210095, China

^2^College of Biotechnology and Pharmaceutical Engineering, Nanjing Tech University, Nanjing 211816, China

^3^Nanchang Key Laboratory of Microbial Resources Exploitation & Utilization from Poyang Lake Wetland, College of Life Sciences, Jiangxi Normal University, Nanchang 330022, China

^†^These authors contributed equally to this work.

**^*^Correspondence:**

Haiyan Ni

E-mail address: [nihaiyan16@163.com](mailto:nihaiyan16@163.com).

Supplementary Material

**Supplementary Table 1** Sensitivity of strain F3Y to antimicrobial agents. ^a^ “+” means that the strain is sensitive to antibiotics, and"-" results in the opposite.

| **Antibiotics (/ piece)** | **Results ^a^** | **Antibiotics (/ piece)** | **Results ^a^** |
| --- | --- | --- | --- |
| Penicillin G (10 μg) | ‒ | Minocycline (5 μg) | ‒ |
| Ampicillin (10 μg) | ‒ | Cefatriaxone (30 μg) | + |
| Kanamycin (30 μg) | ‒ | Neomycin (30 μg) | + |
| Erythromycin (15 μg) | ‒ | Tetracycline (30 μg) | ‒ |
| Gentamicin (10 μg) | + | Cefradine (30 μg) | ‒ |
| Cefuroxime (30 μg) | ‒ | Amikacin (30 μg) | ‒ |

**Supplementary Table 2** List of F3Y‒induced metabolites of oxyfluorfen.

| **Compound** | **Name** | **Molecular Formula** | **Mass spectrum (m/z)** |
| --- | --- | --- | --- |
| oxyfluorfen | oxyfluorfen | C_15_H_11_ClF_3_NO_4_ | 362.04 [M+H]^+^ |
| M1 | *N*‒acetylaminooxyfluorfen | C_17_H_15_ClF_3_NO_3_ | 374.07 [M+H]^+^ |
| M2 | aminooxyfluorfen | C_15_H_13_ClF_3_NO_2_ | 332.06 [M+H]^+^ |
| M3 | 3‒ethoxy‒4‒nitrophenol | C_8_H_9_NO_4_ | 184.06 [M+H]^+^ |
| M4 | 4‒ethoxy‒5‒nitrobenzene‒1,2‒diol | C_8_H_9_NO_5_ | 217.08 [M+NH_4_]^+^ |
| M5 | *N*‒(2‒ethoxy‒4,5‒dihydroxyphenyl)acetamide | C_10_H_13_NO_4_ | 230.10 [M+NH_4_]^+^ |
| M6 | *N*‒(2‒ethoxy‒4‒hydroxyphenyl)acetamide | C_10_H_13_NO_3_ | 213.12 [M+NH_4_]^+^ |
| M7 | 4‒amino‒3‒ethoxyphenol | C_8_H_11_NO_2_ | 171.11 [M+NH_4_]^+^ |
| M8 | (2‒ethoxy‒4,5‒dihydroxyphenyl)carbamic acid | C_9_H_11_NO_5_ | 231.09 [M+NH_4_]^+^ |
| M9 | ((*2E,4E*)‒4‒ethoxy‒1,6‒dihydroxyhexa‒2,4‒dien‒3‒yl) carbamic acid | C_9_H_15_NO_5_ | 240.08 [M+Na]^+^ |
| M10 | (*2E,4E*)‒3‒acetamido‒4‒ethoxyhexa‒2,4‒dienedioic acid | C_10_H_13_NO_6_ | 261.10 [M+NH_4_]^+^ |
| M11 | (*2E,4E*)‒2‒chloro‒4‒(trifluoromethyl)hexa‒2,4‒dienedioic acid | C_7_H_4_ClF_3_O_4_ | 244.98 [M+H]^+^ |

**Supplementary Table 3** Putative genes related to oxyfluorfen degradation in the genome of strain F3Y.

| **Putative name** | **Predictive function** | **Length (bp)** | **Identities** |
| --- | --- | --- | --- |
| *paoY* | phenol‒2‒monooxygenase  (*Micrococcus* sp. JKS001869) | 914 | 99% |
| *paoA* | 1,2‒phenylacetyl‒CoA epoxidase, subunit A  (*Escherichia coli* K12) | 1008 | 94% |
| *paoB* | 1,2‒phenylacetyl‒CoA epoxidase subunit B  (*Escherichia coli* K12) | 303 | 97% |
| *paoC* | 1,2‒phenylacetyl‒CoA epoxidase, subunit C  (*Escherichia coli* K12) | 696 | 81% |
| *paoD* | 1,2‒phenylacetyl‒CoA epoxidase, subunit D  (*Escherichia coli* K12) | 321 | 62% |
| *paoE* | 1, 2‒phenylacetyl‒CoA epoxidase, subunit E  (*Escherichia coli* K12) | 1170 | 57% |
| *paoF* | enoyl‒CoA hydratase  (*Clostridium acetobutylicum* ATCC 824) | 786 | 54% |
| *paoG* | enoyl‒CoA isomerase  (*Bacillus subtilis* 168) | 786 | 51% |
| *paoH1* | 3‒hydroxybutyryl‒CoA dehydrogenase  (*Micrococcus luteus*) | 888 | 59% |
| *paoH2* |  | 933 | 99.68% |
| *paoI* | hydroxyphenylacetyl‒CoA thioesterase  (*Actinobacteria*) | 417 | 77% |
| *paoK* | phenylacetate‒coenzyme A ligase  (*Azoarcus evansii*) | 1350 | 72% |
| *paoZ* | bifunctional aldehyde dehydrogenase/ enoyl‒CoA hydratase/ ether hydrolase  (*Escherichia coli* K12) | 3123 | 64% |
